# Where’s Wanda? The influence of visual imagery vividness on visual search speed measured by means of hidden object pictures

**DOI:** 10.3758/s13414-022-02645-6

**Published:** 2023-01-10

**Authors:** Merlin Monzel, Martin Reuter

**Affiliations:** 1 Department of Psychology, Personality Psychology and Biological Psychology, University of Bonn, Kaiser-Karl-Ring 9, 53111 Bonn, Germany; 2 Center for Economics and Neuroscience (CENs), Laboratory of Neurogenetics, University of Bonn, Bonn, Germany

**Keywords:** Aphantasia, Mental imagery, Real-world visual search, Vividness of visual imagery

## Abstract

Previous research demonstrated effects of visual imagery on search speed in visual search paradigms. However, these effects were rather small, questioning their ecological validity. Thus, our present study aimed to generalize these effects to more naturalistic material (i.e., a paradigm that allows for top-down strategies in highly complex visual search displays that include overlapping stimuli while simultaneously avoiding possibly confounding search instructions). One hundred and four participants with aphantasia (= absence of voluntary mental imagery) and 104 gender and age-matched controls were asked to find hidden objects in several hidden object pictures with search times recorded. Results showed that people with aphantasia were significantly slower than controls, even when controlling for age and general processing speed. Thus, effects of visual imagery might be strong enough to influence the perception of our real-life surroundings, probably because of the involvement of visual imagery in several top-down strategies.

## Introduction

Recently, Monzel et al. (2021) were able to show that visual imagery influences visual search performance. People with aphantasia (= absence of voluntary mental imagery; Monzel et al., 2022; Zeman et al., 2015) and controls were asked to generate mental images before searching the corresponding visual stimuli in a search display. As expected, people with aphantasia were slower in finding the corresponding images than controls, whereas in a verbal control task, no differences occurred. This finding was interpreted as the result of mental imagery priming, a process in which mental imagery changes the response to subsequently presented external stimuli. Another example for mental imagery priming is the binocular rivalry task developed by Pearson et al. (2008), which is often used to assess mental imagery strength (Keogh & Pearson, 2018; Shine et al., 2015). In binocular rivalry, the participants’ eyes are presented with two different images at the same time, whereupon one image becomes dominant and the other one is suppressed. Pearson et al. (2008) were able to show that preceding mental imagery can prime subsequent binocular rivalry dominance (i.e., the probability of perceiving one of the two images shifts in the direction of the previously imagined stimulus). Moreover, the achieved priming scores are stable enough to correlate with other measures of mental imagery, such as the *Vividness of Visual Imagery Questionnaire* (VVIQ; Pearson et al., 2011) and pupil dilation to imagined light (Kay et al., 2022).

While effects of mental imagery have thus been demonstrated in different artificial paradigms, it is questionable to what extent this effect may manifest in everyday life, especially in individuals with aphantasia who cannot benefit from, for instance, enhanced visual search speed through mental imagery (Monzel et al., 2021). Effects of mental imagery seem to be rather small even in laboratory settings (Pounder et al., 2022), which is why the various confounding variables in the field could completely nullify the effect and render it ecologically irrelevant. The aim of the present study was therefore to investigate the effects of mental imagery priming with more naturalistic material. In order to still have the advantages of a standardized measurement, a compromise between computer paradigm and real-world visual search was sought. We tried to meet the following criteria:

1. searching for previously presented stimuli to allow for target templates (Malcolm & Henderson, 2010) that are also accessible in real world visual search (in contrast to Monzel et al., 2021, who used word cues instead of image cues),
2. highly complex visual search displays (in contrast to Monzel et al., 2021, who displayed a 1 × 2 search display with a pool of only three possible distractors),
3. overlaps between the search stimuli since distractors can also (partially) obscure target stimuli in the real world (Alexander & Zelinsky, 2018; Plomp et al., 2004; in contrast to Monzel et al., 2021, who displayed no overlaps), and
4. no influence of instructions on the chosen search strategy (in contrast to Monzel et al., 2021, who encouraged the generation of mental imagery).

We decided to use hidden object pictures often found in children’s books as search displays, as these have practical application in the reality of many people's lives (whether as children or parents) and they meet all the criteria mentioned above while search time is still able to be assessed automatically. First, hidden object pictures familiarize readers with the object which has to be found in a hidden picture to allow for target templates. Second, they present complex scenes with many distractors. Third, the target is often obscured by distractors, and fourth, the presentation of the hidden object pictures has a great prompting character, which makes the presentation of concrete search instructions unnecessary. In line with Monzel et al. (2022), we hypothesized that people with aphantasia would take longer to find the objects hidden in the hidden object pictures, regardless of the fact that participants were not asked to visualize the objects beforehand. Therefore, we aim to show, that mental imagery priming can influence everyday life even without prompting its use.

## Method

### Participants

A total of 104 participants with aphantasia (VVIQ ≤ 23; criterion according to Zeman et al., 2020) and 104 gender (63.0% male, 33.2% female, 3.8% other) and age-matched (*M* = 31.66, *SD* = 11.95) control participants (VVIQ > 23) were recruited via the database of the *[project name; has to be inserted later due to masked review]*. Education was generally high (14.4% primary school or below, 22.6% secondary school, 18.8% A-levels, 44.2% university degree). People with aphantasia (*M* = 114.87, *SD* = 18.39) and controls (*M* = 117.58, *SD* = 15.48) did not differ in intelligence, *t*(106) = 0.80, *p* = .423.

### Procedure

**Fig. 1:**
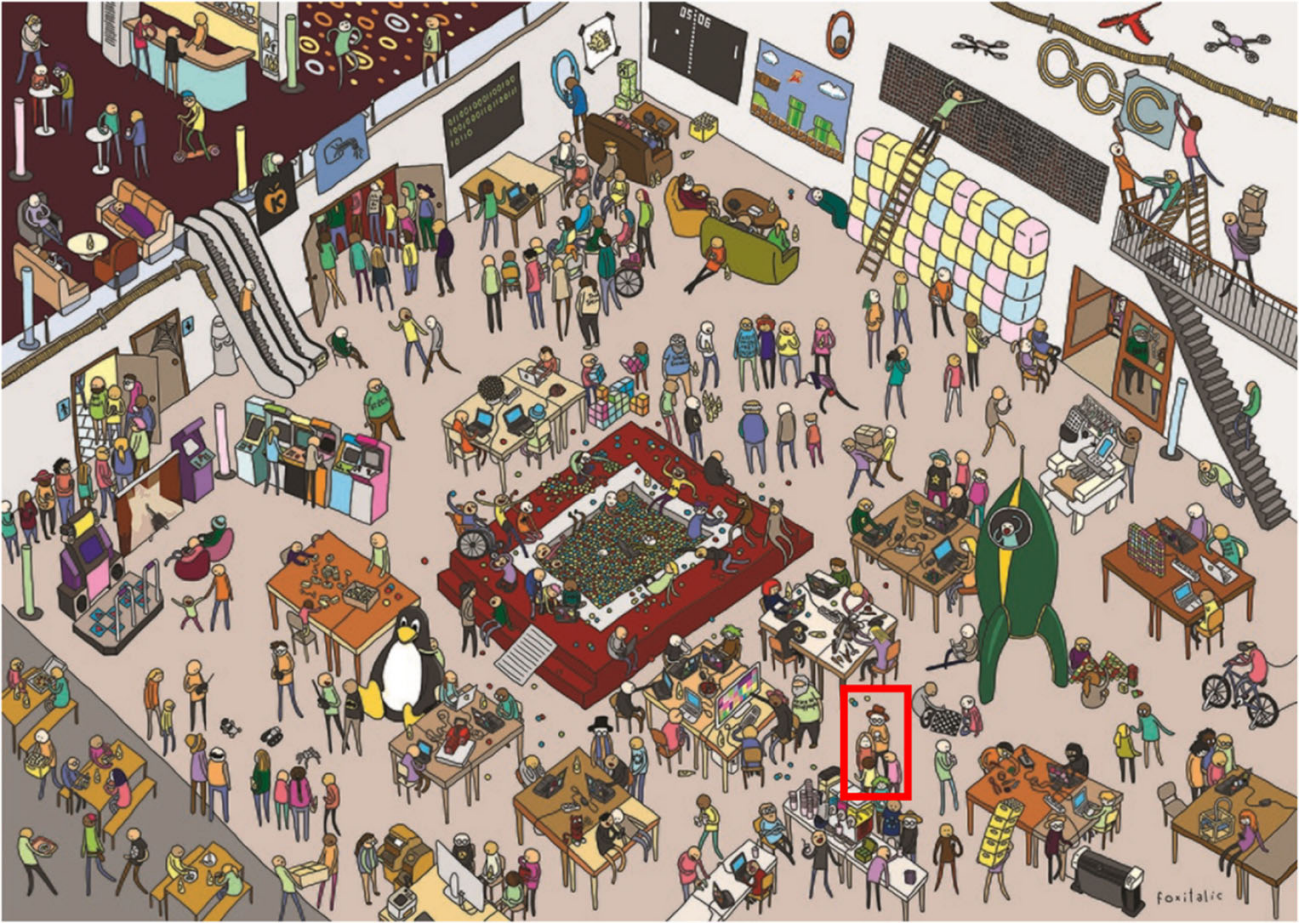
Example of the hidden object pictures including the hidden object (red bordered). File name in Wikimedia Commons: “31c3-Wimmelbild.jpg”. License: Creative Commons Attribution 3.0 Unported. Author: Caro Wedekind. (Color figure online)

The experiment was conducted between August 26, 2021, to October 25, 2021, via the online platform SoSci Survey (Version 3.2.30; Leiner, 2021). Informed consent was obtained in accordance with the World Medical Association Declaration of Helsinki (World Medical Association, 2013). Examination language was English. Participants were asked to run the experiment in a quiet environment. After answering questions about their demographics, they completed the VVIQ (Marks, 1973), a standard questionnaire to assess vividness of visual imagery which was validated in many studies before (e.g., Pearson et al., 2011; Rademaker & Pearson, 2012). Thereafter, among other tasks analyzed elsewhere, the visual search task was displayed, which comprised of six trials. Each trial started with the presentation of the target object until the participant clicked ‘next’. Afterwards, a hidden object picture with the dimensions 600 × 800 pixels was presented (see Fig. 1). In each trial, time measurement started with the presentation of the hidden object picture and ended as soon as the participants clicked on the target object. Finally, the mini-q (Baddeley, 2013) was administered, a speed-based intelligence test to control for general processing speed.

### Statistical analyses

Reaction time differences between participants with aphantasia and controls were examined using a generalized linear mixed-effect model (GLMM) with age and intelligence as *z*-transformed covariates to control for general processing speed. An inverse Gaussian distribution (raw RT, identity link) was used to account for the skewed distribution of reaction times (Lo & Andrews, 2015). Furthermore, nested GLMMs with increasing complexity were performed with step-by-step inclusion of *z*-transformed VVIQ scores, age and mini-q scores as predictors to examine the influence of visual imagery vividness on visual search speed beyond age and general processing speed. For this purpose, the total processing time of the mini-q was divided by the number of points achieved, so that low values correspond to a fast processing time per point. Finally, all analyses were repeated with the exclusion of outliers that fell outside 2 or 3 standard deviations from the mean to control for potential distorting influences of very long reaction times, which could have been caused, for example, by distractions or interruptions.

Moreover, in order to be able to exclude the influence of the target encoding time as a potential confounder, a *t*-test for the time spent on the target presenting page was calculated between the groups as well as a correlation between the search time and the time spent on the target presenting page and a correlation between the VVIQ score and the time spent on the target presenting page. For this, participant who spent more than 5 minutes on the target presenting page were excluded (*N* = 10), as it is likely that these people had left their seats at this point (e.g., to get a glass of water).

## Results

**Fig. 2:**
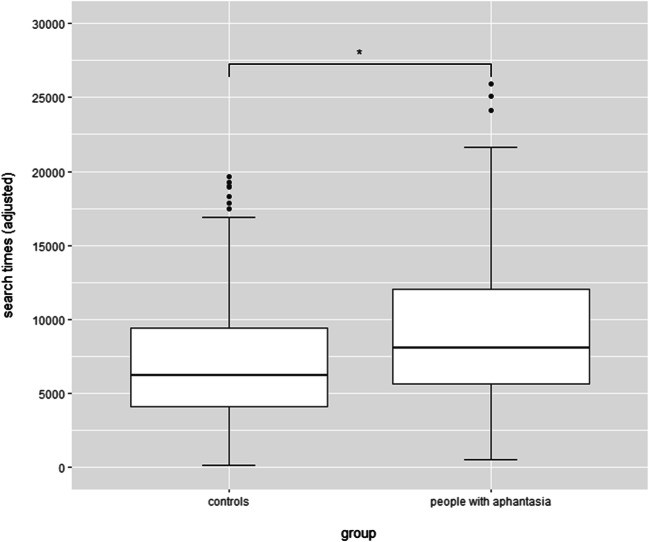
Boxplots of adjusted search times separated by group (in ms)

On average, people with aphantasia (*M* = 8,950 ms, *SE* = 1,905 ms) were 1,488 ms slower than controls (*M* = 7,462 ms, *SE* = 1,926 ms), *F*(1, 104.32) = 6.64, *p* = .011, *d* = 0.20, 95% CI [0.09, 0.31] (see Fig. 2). The coefficients of the hierarchical GLMMs are depicted in Table 1. Vividness of visual imagery was negatively associated with search time, confirming the hypothesis that imagery vividness facilitates visual search. Furthermore, age was positively associated with search speed in Model 2, indicating that participants slow down with age. In Model 3, the age effect disappeared, most likely due to shared variance with general processing speed, *r*(644) = .33, 95% CI [.26, .40], *p* < .001. General processing speed was positively associated with search speed, indicating that people who are generally slower than others also need more time to solve the hidden object picture. Comparisons between the models showed that each model explained significantly more variance than the model before. All effects remained the same after controlling for outliers that fell outside 2 or 3 standard deviations from the mean.

**Table 1:** Nested GLMMs of imagery vividness, age, and processing speed on visual search response times

Variable	β	95% CI for β		*R*^2^	Δ*R*^2^	AIC	BIC
		*LL*	*UL*				
Step 1				.018	.018***	–364.28	–341.93
Vividness of Mental Imagery (VVIQ)	–.14***	–.21	–.06				
Step 2				.026	.008*	–367.41	–340.59
Vividness of Mental Imagery (VVIQ)	–.13**	–.21	–.05				
Age	.09*	.01	.17				
Step 3				.042	.016***	–376.40	–345.11
Vividness of Mental Imagery (VVIQ)	–.13**	–.21	–.05				
Age	.05	–.03	.13				
General Processing Speed (mini-q)	.14**	.06	.21				

*CI* confidence interval*; LL* lower limit*; UL* upper limit*; AIC* Akaike information criterion; *BIC* Bayesian information criterion; *VVIQ* Vividness of Visual Imagery Questionnaire

*p < .05, **p < .01, ***p < .001

Moreover, there were no correlations between the VVIQ score and the target encoding time, *r*(196) = .08, *p* = .256, as well as the search time and the target encoding time, *r*(196) = .13, *p* = .077, excluding the target encoding time as a potential confounder. Time spent on the target presenting page did not differ significantly between participants with aphantasia (*M* = 57.18 s, *SD* = 35.29 s) and controls (*M* = 62.38 s, *SD* = 43.91 s), *t*(196) = 0.92, *p* = .358, *d* = 0.13.

## Discussion

The results show that people with aphantasia are on average slower in finding a hidden object in a hidden object pictures than controls, independent of target encoding time. In fact, the effect of vividness of visual imagery on visual search speed was larger than the effect of age and as large as the effect of general processing speed. Thus, it is shown that the effect of imagery vividness on visual search found by Monzel et al. (2021) can be generalized to more naturalistic material—that is, to paradigms where no search strategies are given via instructions, search displays are more complex, target templates exist (Malcolm & Henderson, 2010) and search stimuli can overlap (Alexander & Zelinsky, 2018; Plomp et al., 2004). One interpretation for these results is enhanced attentional guidance through visual imagery priming, where visual imagery exhibits its influence as a top-down strategy to filter relevant information and to suppress irrelevant information from awareness (e.g., Pearson et al., 2008). However, an alternative approach could be the influence of visual imagery on the decision-making process whether a fixated object is a target or not (Hout & Goldinger, 2014; Yu et al., 2022). Although these two processes are not separable in the context of our study, we were able to show that visual imagery ability influences perception and that individual differences in visual imagery ability might lead to different perceptions of our surrounding world despite the same bottom-up information. In the future, perceptual phenomena should therefore be investigated more closely in connection with mental imagery, such as sensory sensitivity (Dance et al., 2021) and anomalous perception (Königsmark et al., 2021), which have recently been shown to be reduced in aphantasia.

Although we have taken efforts to make our study material more naturalistic compared to the pre-study (Monzel et al., 2021), some limitations regarding the applicability to real life visual search have to be considered. For top-down strategies of visual search (Malcolm & Henderson, 2010), not only target templates play an important role, but also the scene context, which allows us to estimate the probability that a certain object will appear at a certain place within the scene (Boettcher et al., 2018; Võ et al., 2019). For example, it is more likely that our keys will be near the entrance door than in the bathroom sink. While our hidden object pictures provided at least some scene context within the framework of physical laws (e.g., the target could not appear inside a wall), besides that, our targets could appear nearly anywhere independent of real-life regularities. Therefore, future research should include scene context in the investigation of visual imagery effects on visual search (e.g., images of a kitchen, a bedroom, or bathroom) to allow for real life heuristics. Nevertheless, our study was able to generalize the effects of visual imagery to visual search including target templates, free choice of search strategy, complex search displays and overlapping stimuli.

## Conclusion

While mental imagery has long been considered a universal ability by the general population, we know by now that mental imagery ability varies widely between people up to the complete absence of mental imagery (Monzel et al., 2022; Zeman et al., 2015). The present study shows that these differences in mental imagery affect the way how people perceive the world, which might ultimately be useful in enhancing mutual understanding and preventing misunderstandings in social interactions. Especially in people with aphantasia and hyperphantasia (= extremely vivid mental imagery), differences in experiences and behaviour are expected that should be investigated in the future more thoroughly.

## Data Availability

The data and code for all experiments are available at https://osf.io/dc3tf/?view_only=5f11d853d73845d3ba0e3ddde64342a9. None of the experiments was preregistered.
